# Incidence of schizophrenia among migrants in the Netherlands: a direct comparison of first contact longitudinal register approaches

**DOI:** 10.1007/s00127-016-1310-8

**Published:** 2016-11-15

**Authors:** Simon J. Hogerzeil, Albert M. van Hemert, Wim Veling, Hans W. Hoek

**Affiliations:** 1 Parnassia Psychiatric Institute, The Hague, The Netherlands; 2 Department of Psychiatry, Leiden University Medical Center, Leiden, The Netherlands; 3 University Medical Center Groningen, Groningen, The Netherlands; 4 Department of Epidemiology, Mailman School of Public Health, Columbia University, New York, NY USA

**Keywords:** Schizophrenia, Incidence, Migrants, First-contact design, Case register

## Abstract

**Purpose:**

To estimate the effect of selective sampling on first contact (FC) studies of the relation between migration and schizophrenia.

**Methods:**

We compared the FC method directly with a more inclusive longitudinal psychiatric register (LPR) method, by letting both methods estimate age and sex adjusted incidence rate ratios (IRR) in the population of The Hague aged 20–54 years, for the three largest migrant groups (first and second generation Caribbean, Turkish, and Moroccan) relative to the native Dutch population.

**Results:**

Both methods found that the adjusted IRR was higher for migrants than for native Dutch [all migrants IRR = 1.70 (95% Cl 1.30–2.21) for the LPR method and 1.91 (95% Cl 1.15–3.25) for the FC]. The IRR for Moroccans was significantly lower in the LPR [IRR 2.69 (95% 2.10–3.41)] than in the FC study [4.81 (3.41–6.68)]. The FC method was relatively more inclusive for migrants presenting at earlier ages or with shorter durations of prior treatment (DPT) than the native Dutch. This resulted in differential sampling and artificially higher IRRs for Moroccan and, to a lesser extent, Turkish migrants.

**Conclusion:**

We confirm that the incidence of schizophrenia is raised twofold for migrants compared to nonmigrants. Using the LPR method, however, IRR estimates were less pronounced for most migrant groups than in a high quality FC study conducted in the same population. The FC method may overestimate the risk of schizophrenia for migrant groups who seek first mental health at a relatively younger age, or who present directly with schizophrenia.

**Electronic supplementary material:**

The online version of this article (doi:10.1007/s00127-016-1310-8) contains supplementary material, which is available to authorized users.

## Introduction

### Background

Researchers have traditionally used the first contact (FC) method [1] to examine the relation between migration and first episodes of schizophrenia (FES) or first episodes of psychosis (FEP); they used either the WHO’s original FC design [2], later variants that allowed for prior contacts with mental health services) [3–5], or psychiatric registers restricted fully [6, 7] or mainly [8, 9] to first admissions.

A worldwide meta-analysis of studies using the FC method and published between 1977 and 2008 estimated the overall incidence rate ratio (IRR) of schizophrenia at 2.1 (95% 1.8–2.4) for first generation migrants and at 2.4 (95% 2.0–2.9) for second generation migrants, compared to nonmigrants [10]. Very high IRRs were reported in the UK for Black Caribbean [first generation IRR 3.9 (3.4–4.6), second generation 5.8 (3.5–2.4)] and Black Africans [first generation IRR 4.3 (2.8–6.8), second generation 3.7 (2.2–6.3)], and in the Netherlands for Moroccans [first generation IRR 4.0 (2.5–6.3), second generation 5.8 (2.9–11.4)] [11].

We have reported that the FC method can seriously underestimate the incidence of schizophrenia. Using a longitudinal psychiatric register (LPR) to estimate the incidence of schizophrenia, we found that up to two thirds of incident cases had not been included in a FC study conducted in the same population and time frame [1]. Subjects had been missed in the FC study because they were no longer prototypical ‘first contact’ by the time they met criteria for schizophrenia, and at that point were not actively monitored within the FC design anymore (e.g. two thirds had been treated for more than five years before the onset of psychosis, or were aged 40 or older at the time of diagnosis).

### Objective

If the FC method misses two thirds of the schizophrenia onsets, it is logical to ask whether prior findings in FC samples are true for all onsets of schizophrenia, or only for the subset detected by the FC method.

For example, selective sampling could distort FC studies if one population has systematically shorter or longer pathways to the index diagnosis than the other.

In the present study we compared the FC and LPR methods directly in the same study population over the same period to estimate the effect of selective sampling on first contact (FC) studies of the relation between migration and schizophrenia.

We restricted our study to schizophrenia to allow for a direct comparison with a FC study [3], which reported schizophrenia IRs, and as a logical next step from an earlier incidence study by our group [1], which used exactly the same population and comparison.

## Methods

### Case finding with the LPR method

The LPR method to estimate the incidence of schizophrenia has been described elsewhere [1]. In short, the LPR of The Hague is a data warehouse uploaded from the patient registration systems of the Parnassia Psychiatric Institute. It includes virtually all inpatient-, outpatient-, day- and psychiatric residential care, emergency services, and collaborative services for all municipal police stations and a large number of general practitioners. Almost all subjects with psychotic disorders in the city of The Hague are treated at Parnassia and are listed in the LPR. The LPR contains information on date of birth, countries of birth of patients and their parents, successive postal codes, DSM-IV diagnoses and all service contacts for each patient treated at Parnassia from 1997 onwards. Historical (but less complete) records are searchable back to 1980 to identify patients treated before 1997. Diagnoses are recorded at intake and are audited on a regular basis at case conferences, upon internal referrals and when treatment is completed. They are classified according to the DSM-IV under supervision of either a psychiatrist or clinical psychologist.

To calculate the IR and IRR with the LPR, we examined diagnostic histories of all subjects with any service contact with Parnassia in 1980–2009 (*n* = 249,409). We defined the onset of schizophrenia (numerator) as subjects who received a first LPR diagnosis of schizophrenia (DSM-IV 295.x) during the five-year study period 2000–2005, and who resided in The Hague and were aged 20–54 (the age range covered by both methods) at the time of the index diagnosis.

### Case finding with the FC method

The FC method has been described elsewhere [1, 3]. We used individual level data from a first-contact study previously conducted in the same catchment area to calculate incidence rates (IR) and ratios (IRR). The original study used a FC sampling frame to estimate the incidence of all psychoses, excluding psychoses related to somatic disorders or substance abuse. Patients with schizophreniform or schizoaffective disorder were merged into the schizophrenia category. In the original study, 364 residents of the catchment area had been identified with a first psychosis in the age bracket 20–54 during the five-year period 2000–2005. For the comparison in our study, we used only the subset of 254 subjects diagnosed with schizophrenia (i.e. DSM-IV codes 295.x).

### Calculation of the incidence rates and ratios

The same denominators and the same formula of IR and IRR were used for the FC estimate and the LPR.

We used detailed data from the municipality to calculate the number of person years (denominator of the incidence rate). Annual registration data were available for the population of The Hague aged 20–54 years over the five year study period (*n* = 233,803 in 2000, increasing to *n* = 250,671 in 2005); the total person years of observation in the study was 1,221,486.

We used the classification of ethnicity of The Netherlands’ Bureau of Statistics, i.e. Dutch ethnicity is assigned to citizens who are Dutch-born and whose parents were also born in The Netherlands (hereafter referred to as Dutch). If a citizen, or (one of) his or her parents, was born abroad, he or she is assigned to the group of people born in that country. If the parents were born in different foreign countries, the country of birth of the mother determines the assignment to a particular group. In the Netherlands foreign countries of birth are condensed into six categories: (1) Morocco, (2) Surinam, (3) Netherlands Antilles, (4) Turkey, (5) Western or westernized countries (northern, southern or western Europe, the former Yugoslavia, the USA, Canada, Australia, New Zealand, Japan or former Netherlands East Indies) and (6) all other (non-western) countries. For this study we merged categories (2) and (3) into the group ‘Caribbean’ and categories (5) and (6) into the group ‘Other’. Information about first versus second generation status and socioeconomic status (e.g. income level, employment, or level of education) was not reliably available in the LPR data, and was therefore, not included in the analysis. We defined the IR for schizophrenia as the number of treated incident cases per 100,000 person years in the study population. We calculated unadjusted IRs and IRRs for each method, for the three migrant groups relative to the native Dutch. We adjusted the estimates for age and sex by applying the same Poisson regression model to both datasets.

### Comparison of treatment pathways of onsets identified by each method, for each migrant group separately

We compared treatment pathways of onsets identified by each method, for each migrant subpopulation separately. To compare both methods accurately, we excluded onsets listed in the FC who were never listed in the LPR, and corrected for spurious effects from delays in registration. Among citizens aged 15–54, the LPR found 843 onsets of schizophrenia. The FC study found 254 onsets; the subset used for the comparison consisted of 213 subjects ‘identified by both methods’ and 665 additional subjects ‘identified only by the LPR during the study period’; for a detailed account, see the results section in [1].

We defined the duration of prior treatment (DPT) as the interval between first contact with mental health services for any mental disorder and the index diagnosis of schizophrenia, in years.

### Sensitivity analyses

We reported previously that inmigration of identified patients or problems with validity of the clinical diagnoses used in the LPR were likely to be small [1]. Briefly, 95% of LPR cases had resided in the catchment area for six months or longer before being diagnosed with schizophrenia, with a median duration of residence of at least 6.7 years (IQR 2.2–21.7). More than 90% of incident diagnoses listed in the LPR had been audited and confirmed by schizophrenia specialists, or were in fact research diagnoses. Index diagnoses were audited yearly (IQR 0.7–1.2 years), and the 5-year diagnostic stability was 90% or higher.

For this study, we performed additional sensitivity analyses for each migrant group separately to examine differentials in inmigration or diagnostic validity between the subpopulations.

### Statistical analyses

All statistical analyses were conducted in R version 3.2.4 with the packages ‘epitools’, ‘qcc’ and ‘ggplot2’. Confidence limits for the IR and IRR were based on the Poisson distribution, using a mid-P exact test [12]. We used Fisher’s exact test for count data to compare proportions. We modelled the incidence rates of schizophrenia with a generalized linear model using a log link and a quasi-poisson family (i.e. estimating the dispersion parameter from the data to adjust for over-dispersion).

## Results

### Comparison of the two methods’ estimates of incidence rates and -ratios

Table 1 shows adjusted and unadjusted IR and IRR of schizophrenia for each migrant group, for the LPR and FC methods separately. The unadjusted IRR for all migrants relative to the native Dutch was 2.10 (1.63–2.73) in the FC study and 1.69 (1.47–1.94) in the LPR. With the exception of the Caribbean group, all IRR estimates for migrants groups were lower in the LPR than in the FC. This difference was statistically significant for Moroccans only, with an age and sex adjusted IRR estimate of 4.81 (95% 3.41–6.68) in the FC study compared to 2.69 (95% CI 2.10–3.41) in the LPR.

**Table 1:** Schizophrenia incidence rates and incidence rate ratios for migrants relative to the native Dutch, for two methods

	PY	Complete data (LPR of The Hague)				First contact sample (Veling et al. 2007)			
		*N*	IR (95% CI)	Unadj. IRR (95% CI)	Adj. IRR (95% CI)	*N*	IR (95% CI)	Unadj. IRR (95% CI)	Adj. IRR (95% CI)
All	1221.486	843	69 (64–74)			254	21 (18–24)		
Native Dutch	659.589	346	52 (47–58)			91	14 (11–17)		
Migrants	561.897	497	88 (81–97)	1.69 (l.47–1.94)	1.70 (1.30–2.21)	163	29 (25–34)	2.1 (1.63–2.73)	1.91 (1.15–3.25)
Caribbean	159.426	180	113 (97–131)	2.15 (1.79–2.57)	2.18 (1.83–2.6)	40	25 (18–34)	1.82 (1.24–2.62)	1.70 (1.19–2.4)
Turkish	77.198	57	74 (56–96)	1.41 (1.06–1.85)	1.40 (1.06–1.83)	26	34 (22–49)	2.45 (1.55–3.74)	1.94 (1.26–2.88)
Moroccan	54.430	77	141 (112–177)	2.70 (2.10–3.44)	2.69 (2.1–3.41)	46	85 (62–113)	6.14 (4.27–8.7)	4.81 (3.41–6.68)
Other	270.843	183	68 (58–78)	1.29 (l.07–l.54)	1.30 (1.09–1.54)	51	19 (14–25)	1.37 (0.96–1.92)	1.32 (0.95–1.82)

*LPR* longitudinal psychiatric register of The Hague, *PY* person-years, *IR* incidence rate per 100,000 person-years, *IRR* incidence rate ratio, relative to the native Dutch, *CI* confidence interval, *unadj* unadjusted, *adj* adjusted for age and sex

When compared with the FC method, the LPR added relatively more cases to the native Dutch category (346 cases in the LPR vs. 91 cases in the FC; 280% more) and relatively fewer cases to the Moroccan category (77 vs 46; 67% more). The resulting larger size of the native Dutch reference category in the LPR estimates reduced the age and sex adjusted IRR slightly for migrants in general (from 2.1 in the FC to 1.9 in the LPR). As the Moroccan group increased much less than the Dutch using the LPR method, their IRR decreased significantly (from 4.81 to 2.69). A similar but less pronounced shift was found for Turkish migrants.

### Comparison of the treatment pathways of onsets included by the two methods

**Fig. 1:**
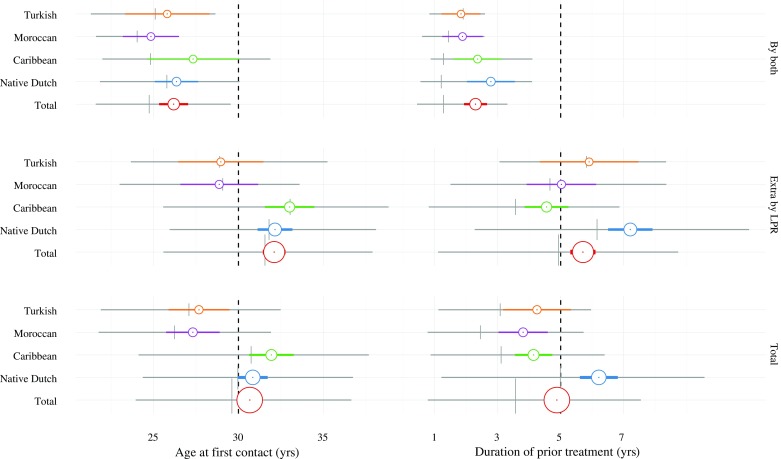
Migrant differentials in pathways to index diagnosis. *Grey horizontal bars* represent the interquartile rate, the *grey crosshair* represents the median; *colored horizontal bars* represent the 95% confidence interval of the mean, *colored bullets* represent the mean; the size of the *colored bullets* and the thickness of the *colored bars* is proportional to the number of cases

Age at first contact and duration of prior treatment are shown in Fig. 1, stratified by migrant group, and by method (cases identified by both methods versus additional cases identified by the LPR). Sociodemographic characteristics and pathway characteristics are given in Supplemental Table 1.

Subjects identified by both methods (*n* = 213) were aged 30 or less at first contact (median 26.2 years; interquartile rate (IQR) 25.3–27.0 years for all subjects), and had been treated for less than five years before the index diagnosis of schizophrenia (DPT = median 2.3 years; IQR 1.9–2.7). In this subset, all migrant subgroups had similar ages at first contact, and duration of treatment.

Among 665 additional cases identified by the LPR the majority had a relatively late onset. Most were aged 30 or older at first contact (median 32.1 years; IQR 31.4–32.8 for all subjects), and had been treated for more than five years before the index diagnosis of schizophrenia (DPT = median 5.7 years; IQR 5.3–6.1). They were mainly Caribbean and native Dutch diagnosed at relatively older ages, and native Dutch with relatively longer durations of prior treatment.

### Sensitivity analysis

Sensitivity analyses indicated that for the Caribbean, Turkish and Moroccan cases, measures of potential inmigration, diagnostic stability and diagnostic validity in the LPR were equivalent to those of the native Dutch (Supplement 2). Nonparametric tests indicated that clinicians were not slower to diagnose psychotic symptoms as schizophrenia (e.g. indefinitely diagnosing ‘psychosis NOS’) with native Dutch than with migrant subpopulations (i.e. no migrant differentials in the interval between initial diagnosis of psychosis (any type other than schizophrenia) and ultimate diagnosis of schizophrenia: Kruskal–Wallis χ^2^ = 6.8164, *df* = 4, *p* value = 0.1459).

## Discussion

Both the FC and the LPR methods found that the age and sex adjusted IRR is significantly higher for all migrant groups compared to the native Dutch [for all migrants IRR 1.70 (95% CI 1.30–2.21) for the LPR method and 1.91 (95% CI 1.15–3.25) for the FC].

The IRR for Moroccans was significantly lower in the LPR [IRR 2.69 (95% 2.10–3.41)] than in the FC study [4.81 (3.41–6.68)]. The IRR estimates in the LPR were lower for the Turkish and higher for the Caribbean than in the FC study, but these shifts were not statistically significant.

### Interpretation

In one population, the FC identified 254 onsets schizophrenia, and the LPR 843 onsets. The onsets identified only by the LPR had a different mix of migrants than the onsets identified by both methods. The LPR method identified a relatively large number of native Dutch and Turkish onsets with a long DPT, and Caribbeans engaging with mental health services at older ages. The FC method identified mostly migrants with earlier onsets (presenting at earlier ages and with shorter DPT than the native Dutch), which in practice resulted in overinclusion of Moroccans and, to a lesser extent, Turkish migrants.

The evidence on the relation between migration and incidence of schizophrenia is nearly exclusively based on the FC sampling frame [1, 13]. Danish register studies [8, 9] have used the LPR method, but in their region had no corresponding FC estimates available for direct comparison. Indirect comparisons of their findings with FC data in other countries [11, 14] are complicated by methodological differences (e.g. other clinical populations, other migrant groupings).

The evidence on migrant differentials in pathways to diagnosis is difficult to interpret because the social, cultural and health service context vary widely between countries [15], and because there is no standardized definition of pathways to- and through mental health services. Prior studies have used overlapping concepts such as ‘access to mental health services’ [16–18], ‘duration of untreated psychosis’ (DUP) [19], ‘negative pathways’ [20] and (in our study) ‘age at first contact with mental health services’ or ‘duration of prior treatment’.

There is some evidence on migrant differentials in pathways through mental health services. Studies from the UK have reported that people from African descent with a first episode of psychosis (FEP) are more likely than other migrant groups to come into contact with mental health services through negative and adversarial routes [15, 21]. Similar findings were later reported for Moroccans and Caribbean in Rotterdam [22] and Amsterdam [23].

Migrant differentials in pathways through services (sometimes resulting in overinclusion in FC samples) may help explain why FC studies report that certain migrant groups have a very high risk of schizophrenia [15, 21, 24, 25]. This might be the case for Moroccans in the Netherlands [3] and Black Africans and Black Caribbean in the UK [5], because these groups are also known to have more negative (and in our study, shorter/earlier) pathways through services, compared to migrants with a lower risk of schizophrenia, and nonmigrants.

Various mechanisms may explain how migration is related both to a higher risk of schizophrenia and to earlier or shorter pathways through services. Higher levels of stress [26, 27], related to factors such as social defeat [28], discrimination [3, 29] or ethnic density [30] may not only increase the lifetime risk of schizophrenia, but also lead to earlier onsets and negative pathways. Such ‘precipitated onsets’ could be mediated by social processes related to culture, stigmatization, or (lack of) social support [20], by causing more dysfunction or modifying the clinical presentation.

Migrant differentials in pathways through care do not necessarily distort schizophrenia IRR estimates, as long as all possible pathways to the index diagnosis are accounted for. This is not a problem for the LPR method. But for some groups in FC studies it may lead to inflated IRR estimates because the FC method over includes groups with early onsets and short DPTs.

### Strengths and limitations

The main strengths of our study are that it was conducted in a well defined urban catchment area with a 45% share of migrants, that the FC study used in the comparison meets the highest quality standards [3, 5, 10, 31], and that the LPR was based on a data warehouse, synchronized every day with data from virtually all mental health services in the catchment area. The longitudinal sampling frame covered all treatment pathways from 1980 to 2009.

Both methods were restricted to treated subjects, and typical limitations of treated incidence studies apply, such as the risk of overinclusion of cases (e.g. due to inmigration of prevalent cases into the catchment area, or diagnostic errors), and the risk of underinclusion (e.g. due to cases avoiding mental health treatment entirely). Sensitivity analyses showed that potential distortions by these factors were likely to be small: very few cases moved into the catchment area shortly before the index diagnosis was made, and the diagnostic process was robust [1].

Migrant differentials in access to mental health care would affect both methods equally, and therefore, cannot account for the differences observed between them; furthermore, surveys of access to care from different countries [16–18] and meta-analyses of DUP-studies [19] reported no systematic differentials.

There is evidence that migrants drop out of mental health treatment more frequently than nonmigrants [32, 33]. Some migrants may have dropped out before the onset of schizophrenia and then been missed by one or both methods. This would deflate the migrant IRR estimate. In the 20–54 working age bracket, access to welfare benefits would be an additional incentive for undiagnosed but disabled schizophrenia patients to reengage with mental health services. These and other cases who reengaged would be listed in the register and ultimately detected as incident cases. They may then have been classified in an older age group.

Cross-cultural diagnostic bias could also have confounded our IRR estimates [34–37]. We did not estimate cross-cultural diagnostic bias directly in the present study. Indirectly, however, we found no migrant differentials in diagnostic validity or stability in either FC or LPR study samples. As noted above, clinicians were not more conservative in diagnosing schizophrenia with native Dutch than with migrant subpopulations.

Unfortunately, we had no reliable data to examine potential confounding from socioeconomic status (SES) at time of onset. In our study (Table 1), the incidence of psychotic disorders for Turkish immigrants was only modestly increased, while they have much lower income, educational and employment levels than Surinamese migrants, whose relative risk was high [38]. In the literature, the strength and nature of the relation between SES and schizophrenia remains unclear [38–41]. In line with two comparable studies [42, 43], we expect that adjusting for individual SES in our data would attenuate the migrants’ IRR estimates but not explain them.

Our findings of overinclusion of subjects presenting at younger ages and/or with shorter duration of prior treatment probably apply to all FC studies of schizophrenia (i.e. first episode of schizophrenia or FES), but we have not shown that it applies to studies of all psychoses (i.e. first episode of psychosis, or FEP).

It seems prudent to assume that selective sampling also occurs in FEP studies. To assume otherwise, for migration as a risk factor, would imply that there are no migrant groups with FEP who present at systematically younger ages, or who have systematically shorter DPT, compared to other migrant groups or to nonmigrants. To our knowledge, this hypothesis has not yet been tested directly.

The indirect evidence is mixed. As noted above, Anderson et al. [15] found that specific migrant groups such as Blacks with FEP had more negative pathways than nonmigrants. High quality FC studies in the UK [4, 14] and in The Hague have reported migrant IRRs for both FES and FEP, and the patterns were similar. Finally, we speculate that overdiagnosis of psychosis among migrants (diagnostic bias) could translate into earlier diagnosis of psychosis among migrants. There is some evidence that diagnostic bias distorts FEP and FES differently [29], but the direction and extent of this difference is unclear.

## Conclusion

Compared to the FC method, the LPR method also found that the incidence of schizophrenia is raised roughly twofold for migrants compared to nonmigrants, but its IRR estimates are less extreme. To the extent that additional cases identified by the LPR method are true incident cases of schizophrenia, LPR estimates are more precise (larger sample, smaller confidence intervals) and possibly more valid (less differential sampling) than FC estimates. Migration is related both to a higher risk of schizophrenia and to specific pathways through services. The FC method may overestimate the risk of schizophrenia for migrant groups who tend to seek first mental health care at young age, or who present directly with schizophrenia.

Our results suggest a new explanation for the very high risk of schizophrenia measured among some migrant groups in FC studies: some migrant populations are found in higher numbers in FC samples not only because they develop schizophrenia more frequently, but also because they follow other pathways through treatment than nonmigrants do.

Other risk factors associated with the pathway to the index diagnosis such as age, gender or socioeconomic factors may also result in differential sampling in FC studies and should also be re-examined.

## Electronic supplementary material

Below is the link to the electronic supplementary material.

Supplementary material 1 (PDF 17 kb)
