# Supplementary material for: Incidence of schizophrenia among migrants in the Netherlands: a direct comparison of first contact longitudinal register approaches
Source: Soc Psychiatry Psychiatr Epidemiol. 2016 Nov 15;52(2):147–54. doi: 10.1007/s00127-016-1310-8 (PMC5329083; doi:10.1007/s00127-016-1310-8)
Supplement: Supplementary file 1 — Supplementary material 1 (PDF 17 kb) [file 127_2016_1310_MOESM1_ESM.pdf]

Table S1: Sociodemographic and pathway characteristics

	By both				Extra by LPR				Total			
Migrant group	N	% male (n)	AFC (sd)	DPT (sd)	N	% male (n)	AFC (sd)	DPT (sd)	N	% male (n)	AFC (sd)	DPT (sd)
Native Dutch	81	74.1 (60)	26.3 (5.8)	2.8 (3.4)	278	65.1 (181)	32.2 (8.7)	7.2 (6)	359	67.1 (241)	30.8 (8.5)	6.2 (5.8)
Caribbean	35	74.3 (26)	27.3 (7.8)	2.4 (2.2)	151	68.9 (104)	33.0 (9.0)	4.6 (4.3)	186	69.9 (130)	31.9 (9.1)	4.1 (4.1)
Turkish	24	70.8 (17)	25.8 (5.9)	1.8 (1.5)	35	74.3 (26)	29.0 (7.3)	5.9 (4.6)	59	72.9 (43)	27.7 (6.9)	4.2 (4.1)
Moroccan	34	76.5 (26)	24.8 (4.7)	1.9 (1.8)	54	79.6 (43)	28.9 (8.4)	5 (4)	88	78.4 (69)	27.3 (7.4)	3.8 (3.7)
Other	39	74.4 (29)	26.2 (7.4)	1.9 (2.5)	147	62.6 (92)	33.0 (8.7)	4.3 (4.4)	186	65.1 (121)	31.6 (8.9)	3.8 (4.2)
Total	213	74.2 (158)	26.2 (6.3)	2.3 (2.7)	665	67.1 (446)	32.1 (8.8)	5.7 (5.2)	878	68.8 (604)	30.7 (8.6)	4.9 (5.0)

By both: incident cases identified by both methods; Extra by LPR: additional cases identified by the longitudinal psychiatric register; AFC (sd): age at first contact with mental health services (standard deviation); DPT (sd): duration of prior treatment before the index diagnosis of schizophrenia (standard deviation)

Table S2: Sensitivity analysis

	Caribbean	Turkish	Morrocan	Other	Native Dutch	All migrants	Total
Number of cases	180	57	77	183	346	497	843
**Indicators of diagnostic validity**							
Years in catchment area before index (95% CI)	9.4 (2.6 to 22.4)	14.1 (4.7 to 22.5)	7.4 (2.9 to 13.8)	4.8 (0.8 to 10.2)	11.1 (3.3 to 27.4)	7.4 (1.5 to 17.1)	8.5 (2.2 to 21.7)
Share LTF or retracted during first year after index (n)	10.6 (19)	3.5 (2)	7.8 (6)	24 (44)	15.9 (55)	14.3 (72)	14.9 (126)
Mean no of audits (95% CI)	4.5 (3 to 6)	5 (3 to 7)	4 (3 to 6)	4 (1 to 6)	5 (2 to 7)	4 (3 to 6)	4 (2 to 7)
Mean no of teams who did audits (95% CI)	2 (1 to 3)	2 (1 to 4)	2 (1 to 3)	2 (1 to 3)	2 (1 to 4)	2 (1 to 3)	2 (1 to 3)
Mean interval between audits, in years (95% CI)	1.1 (0.7 to 1.5)	1.1 (0.8 to 1.4)	1.2 (0.9 to 1.7)	1.1 (0.6 to 1.5)	1 (0.7 to 1.4)	1.1 (0.7 to 1.5)	1.1 (0.7 to 1.5)
5-year stability (95% CI)	89.5 (84.5 to 94.7)	92.7 (85.0 to 1)	88.1 (80.6 to 96.2)	92.8 (88.3 to 97.5)	90.6 (87.1 to 94.2)	90.7 (87.8 to 93.7)	90.6 (88.4 to 92.9)
**Levels of available evidence to support clinical diagnosis of schizophrenia**							
Research diagnosis (%)	34 (20.0)	23 (41.1)	26 (35.6)	42 (25.1)	70 (20.9)	125 (26.8)	195 (24.3)
Very high (%)	52 (30.6)	17 (30.4)	26 (35.6)	55 (32.9)	124 (37)	150 (32.2)	274 (34.2)
High (%)	69 (40.6)	13 (23.2)	20 (27.4)	53 (31.7)	115 (34.3)	155 (33.3)	270 (33.7)
Standard (%)	15 (8.8)	3 (5.4)	1 (1.5)	17 (10.2)	26 (7.8)	36 (7.7)	62 (7.7)
Suspect for in-migration (excluded)	10	1	4	16	11	31	42
**Incidence rates at incremental levels of available evidence**							
Including only research diagnosis (95% CI)	21 (15 to 30)	30 (19 to 45)	48 (31 to 70)	16 (11 to 21)	11 (8 to 13)		
Including also very high quality (95% CI)	54 (43 to 67)	52 (37 to 71)	96 (71 to 125)	36 (29 to 44)	29 (25 to 34)		
Including also high quality (95% CI)	97 (83 to 114)	69 (51 to 90)	132 (104 to 167)	55 (47 to 65)	47 (42 to 52)		
Including also standard quality (95% CI)	107 (91 to 124)	73 (55 to 94)	134 (105 to 169)	62 (53 to 72)	51 (45 to 57)		
Including all cases (95% CI) (even suspect cases)	113 (97 to 131)	74 (56 to 96)	141 (112 to 177)	68 (58 to 78)	52 (47 to 58)		
**Incidence ratios at incremental levels of available evidence**							
Including only research diagnosis (95% CI)	2.0 (1.3 to 3)	2.8 (1.7 to 4.5)	4.5 (2.8 to 7)	1.5 (1 to 2.1)	ref		
Including also very high quality (95% CI)	1.8 (1.4 to 2.4)	1.8 (1.2 to 2.5)	3.3 (2.4 to 4.4)	1.2 (1 to 1.6)	ref		
Including also high quality (95% CI)	2.1 (1.7 to 2.5)	1.5 (1.1 to 1.9)	2.8 (2.2 to 3.6)	1.2 (1 to 1.4)	ref		
Including also standard quality (95% CI)	2.1 (1.7 to 2.5)	1.4 (1.1 to 1.9)	2.6 (2 to 3.4)	1.2 (1 to 1.5)	ref		
Including all cases (even suspect cases) (95% CI)	2.2 (1.8 to 2.6)	1.4 (1.1 to 1.9)	2.7 (2.1 to 3.4)	1.3 (1.1 to 1.5)	ref		
